# Fluorescence Modulation of Conjugated Polymer Nanoparticles Embedded in Poly(*N*-Isopropylacrylamide) Hydrogel

**DOI:** 10.3390/polym13244315

**Published:** 2021-12-09

**Authors:** Ho Namgung, Seonyoung Jo, Taek Seung Lee

**Affiliations:** Organic and Optoelectronic Materials Laboratory, Department of Organic Materials Engineering, Chungnam National University, Daejeon 34134, Korea; namgungho90@cnu.ac.kr (H.N.); oncsy@cnu.ac.kr (S.J.)

**Keywords:** conjugated polymer dots, PNIPAM, thermoreversible fluorescence, hydrogel

## Abstract

A series of conjugated polymers (CPs) emitting red, green, and blue (RGB) fluorescence were synthesized via the Suzuki coupling polymerization. Polymer dots (Pdots) were fabricated by the reprecipitation method from corresponding CPs, in which the Pdot surface was functionalized to have an allyl moiety. The CP backbones were based on the phenylene group, causing the Pdots to show identical ultraviolet-visible absorption at 350 nm, indicating that the same excitation wavelength could be used. The Pdots were covalently embedded in poly(*N*-isopropylacrylamide) (PNIPAM) hydrogel for further use as a thermoresponsive moiety in the polymer hydrogel. The polymer hydrogel with RGB emission colors could provide thermally reversible fluorescence changes. The size of the hydrogel varied with temperature change because of the PNIPAM’s shrinking and swelling. The swollen and contracted conformations of the Pdot-embedded PNIPAM enabled on-and-off fluorescence, respectively. Fluorescence modulation with 20 to 80% of the hydrogel was possible via thermoreversibility. The fluorescent hydrogel could be a new fluorescence-tuning hybrid material that changes with temperature.

## 1. Introduction

The detection of environmental changes has received significant attention. For example, there is much interest in polymeric hydrogels that respond to environmental stimuli because of their potential applications in drug delivery [1,2,3,4,5,6], chemical and biosensing [7,8], adsorption [9], shape-control [10], and color tuning [11,12,13] The physical and chemical properties of these responsive polymer hydrogels can be altered by light, mechanical force, pH, and temperature [14].

Among the hydrogels, poly(*N*-isopropylacrylamide) (PNIPAM) is well known for its excellent thermo responsiveness [15,16]. PNIPAM has a lower critical solution temperature (LCST) of around 32 °C in an aqueous medium. Below LCST, the polymer is water-soluble because its polymer chains are fully extended with random coil conformation. Because of the hydrophobic interaction, PNIPAM shrinks at temperatures above the LCST. This feature renders PNIPAM-based hydrogels suitable for use in drug-delivery systems. We attempted to investigate the relationship between shrinking and swelling and fluorescence changes of fluorescent PNIPAM hydrogels in aqueous solutions. Most fluorescent PNIPAM hydrogels were prepared via copolymerization of PNIPAM with a fluorescent monomer [17], but fluorescent monomers showed lower stability and brightness than conjugated polymer dots (Pdots) [18]. The quantum dots (QDs) in PNIPAM hydrogel were also investigated and QD fluorescence in the hydrogel was changed by LCST [19]. However, QDs had low biocompatibility because of the heavy metal in QDs.

Pdots were derived from conjugated polymers (CPs) and their high fluorescence was inherited from their pristine CPs. In addition, they have various versatile properties, such as uniform dispersion in water and easy surface functionalization [20,21,22,23]. Thus, a number of Pdots with various chemical structures were investigated for their application in chemo- and biosensing [24,25,26,27,28,29,30].

We are reporting on the thermoresponsive fluorescence tuning of Pdots in a PNIPAM-based hydrogel. Thermally responsive fluorescence tuning in the hydrogel was reported using poly(2-isopropyl-2-oxazoline) copolymerized with various fluorescent dyes to attain various emission colors [31]. Graphene oxide was used as a fluorescence quencher to achieve fluorescence tuning according to temperature, in which a PNIPAM copolymer controlled the distance between the quencher and the polymeric dye [32]. However, the use of Pdot-hybridized hydrogel for fluorescence tuning has rarely been reported. To investigate the thermo responsiveness, a hybrid material based on Pdots and PNIPAM was synthesized, in which Pdots were linked with PNIPAM after allyl-group functionalization in a Pdot surface. The Pdots were covalently immobilized in PNIPAM, enabling stable fluorescence modulation without a release from the PNIPAM matrix. The changes in fluorescence intensity, as well as in the hydrodynamic diameter of the hybrid material, were investigated, resulting from the LCST behavior of PNIPAM. Upon heating the hybrid material above the LCST, the fluorescence intensity decreased mainly because of shrinkage of the PNIPAM coils, in which the fluorescence was recovered after cooling to room temperature. The hydrogel-based, fluorescence-tunable material has great potential for various applications in sensing.

## 2. Experimental

### 2.1. Materials and Instrumentation

All chemicals were purchased from Sigma-Aldrich (St. Louis, MO, USA) and solvents were purchased from Samchun Chemicals (Seoul, Korea). All reagents were used without further purification unless otherwise noted. Maleic anhydride and benzoyl peroxide were purified by recrystallization before use. The inhibitor in styrene was removed using an inhibitor remover column (Sigma-Aldrich). The ^1^H NMR and ^13^C NMR data were obtained on a Bruker Fourier-300 spectrometer (Bruker, Karlsruhe, Germany). Elemental analysis (EA) was performed with a CE Instruments EA-1112 elemental analyzer (CE Instruments, Milan, Italy). The Fourier transform infrared (FT-IR) spectra were obtained on a Bruker Tensor 27 spectrometer (Bruker, Karlsruhe, Germany). The ultraviolet-visible (UV-vis) absorption spectra were recorded on a PerkinElmer Lambda 35 spectrometer (PerkinElmer, Waltham, MA, USA). The photoluminescence spectra with variation in temperature were taken using a Varian Cary Eclipse spectrometer (Agilient, Santa Clara, CA, USA) with a PCB-1200 temperature controller. The molecular weights (MWs) of the polymers were determined by gel permeation chromatography (GPC), with tetrahydrofuran (THF) as eluent with a polystyrene standard. Zeta-potentials and size distributions were measured by dynamic light scattering (DLS, Zetasizer Nano ZS, Malvern, Worcestershire, UK). Scanning electron microscopy (SEM) images were obtained using a Hitachi S-4800 instrument (Hitachi, Tokyo, Japan). Transmission electron microscopy (TEM) images and energy-dispersive X-ray spectroscopy (EDS) data were taken using a JEM-3011, JEOL apparatus, Tokyo, Japan.

### 2.2. Synthesis of a Blue-Emitting CP (BCP)

M1 (0.448 g, 0.91 mmol) and M4 (0.330 g, 1 mmol) were dissolved in tetrahydrofuran (THF) containing an aqueous 2 M potassium carbonate solution (3 mL) under argon atmosphere. After addition of tetrakis (tripenylphosphine)palladium (0) (0.0526 g, 0.045 mmol), the reaction mixture was stirred at 100 °C for 30 h. After the reaction, the mixture was cooled and added to methanol (300 mL) and the precipitate was isolated by filtration. The precipitates were extracted with acetone for 24 h in a Soxhlet apparatus to remove oligomers and catalyst residues. After drying under vacuum, a gray powder was obtained (yield 0.15 g, 38%). ^1^H NMR (300 MHz, CDCl_3_): 7.71 (s, 2H), 7.10 (m, 4H), 3.99 (s, 4H), 1.75 (s, 8H), 1.41 (m, 16H), 0.88 (m, 6H) ppm. ^13^C NMR (CDCl_3_): 129.08, 77.32, 77.20, 76.73, 69.60, 40.83, 31.86, 29.39, 26.12, 22.66, 14.10 ppm. FT-IR (KBr pellet, cm^−^^1^): 2924-2852 (C-H), 1469 (C=C), 1209 (aromatic C-H) Anal Calcd. For C_30_H_46_O_2_: C, 82.14%; H, 10.57%. Found. C, 81.72%; H, 9.76%.

### 2.3. Synthesis of a Green-Emitting CP (GCP)

M1 (1.05 g, 2.1 mmol), M2 (0.07 g, 0.24 mmol), and M4 (0.86 g, 2.6 mmol) were dissolved under an argon atmosphere in a mixture of THF containing an aqueous 2 M potassium carbonate solution (5 mL). The subsequent procedure was identical to that used for BCP. After drying under vacuum, a gray powder was obtained (yield 0.74 g, 80%). ^1^H NMR (300 MHz, CDCl_3_): 7.71 (s, 3.78 H), 7.10 (m, 2.22 H), 3.99 (s, 3,56 H), 1.76 (s, 7.12 H), 1.48 (m, 7.12 H), 1.27 (m, 7.12 H), 0.87 (m, 5.34) ppm. ^13^C NMR (CDCl_3_): 150.48, 129.07, 77.86, 77.20, 76.68, 69.60, 31.82, 29.89, 26.12, 22.67, 14.10 ppm. FT-IR (KBr pellet, cm^−^^1^): 2924-2852 (C-H), 1610 (C=N), 1492 (C=C), 1207 (aromatic C-H) Anal Calcd. For C_26.5_H_36.5_N_0.2_O_1.8_: C, 82.23%; H, 10.23%; N, 0.79%. Found. C, 81.14%; H, 9.51%; N, 0.78%.

### 2.4. Synthesis of a Red-Emitting CP (RCP)

M1 (0.47 g, 0.95 mmol), M3 (0.19 g, 0.41 mmol), and M4 (0.45 g, 1.36 mmol) were dissolved under an argon atmosphere in a mixture of THF containing an aqueous 2 M potassium carbonate solution (3 mL). The subsequent procedure was identical to that used for BCP (yield 0.78 g, 73%). ^1^H NMR (300 MHz, CDCl_3_): 8.15 (s, 1.26 H), 7.7 (m, 2.2 H), 7.6 (m, 0.74 H), 7.4 (m, 0.74 H), 7.1 (s, 0.74 H), 7.0 (m, 0.74 H), 6.9 (m, 0.74 H), 2.03 (t, 2.52 H), 1.85 (m, 5.04 H), 1.72 (s, 5.04 H), 1.51 (s, 5.04 H), 0.87 (d, 3.78 H) ppm. ^13^C NMR (CDCl_3_): 77.44, 77.22, 76.59, 31.82, 29.71, 22.67, 14.12 ppm. FT-IR (KBr pellet, cm^−^^1^): 2922-2852 (C-H), 1737 (C-O), 1608 (C=N), 1485 (C=C), 1253 (C=S), 1205 (aromatic C-H) Anal Calcd. For C_25.0_H_28.9_N_0.7_O_1.3_S_1.1_: C, 75.88%; H, 7.29%; N, 2.61%; S, 8.97%. Found. C, 74.01%; H, 7.31%; N, 2.16%; S, 8.13%.

### 2.5. Synthesis of Poly(Styrene-Co-Maleic Anhydride) (PSMA)

Maleic anhydride (2.94 g, 0.03 mmol), styrene (3 mL, 0.0258 mmol), and dibenzoyl peroxide (BPO, 0.03 g, 0.12 mmol) were dissolved in toluene (40 mL) at room temperature under an argon atmosphere. The solution was stirred at 80 °C for 2 h. After the polymerization, the mixture was cooled to room temperature and poured in methanol (200 mL). The precipitates were isolated by filtration and washed with methanol three times. After drying under vacuum, a white powder was obtained (yield 5.2 g, 92%). ^1^H NMR (300 MHz, CDCl_3_): 7.4 (s, 0.53 H), 7.2 (m, 2.12 H), 3.3 (s, 0.94 H), 2.8 (s, 1.06 H), 2.3 (m, 0.53 H) ppm. FT-IR (KBr pellet, cm^−^^1^): 3030–2925 (C-H), 1857–1780 (C=O), 1496–1454 (C=C), 1222 (C-O-C). Anal Calcd. For C_6.04_H_5.06_O_1.47_: C, 71.64%; H, 5.00%. Found. C, 70.13%; H, 5.01%.

### 2.6. Fabrication of Polymer Nanoparticles (NPs)

Each CP (BCP, GCP, and RCP) was dissolved in THF (1 mg/mL). PMAS was dissolved separately in THF (1 mg/mL). The CP (250 μL) and PSMA solutions (150 μL) were added to THF (4.65 mL). After complete dissolution, the solution was quickly injected into water (10 mL) under sonication. After 10 min sonication, THF was removed by nitrogen blowing and the mixture was concentrated to 5 mL at 75 °C. During nitrogen blowing, carboxylic acid was generated on the NP surface. After filtration with a 0.2 μm filter, nanoparticle solution was obtained. The polymer nanoparticles (Pdots) were denoted as BPdots, GPdots, and RPdots from their pristine BCP, GCP, and RCP, respectively.

### 2.7. Pdots Coated with Allyl Amine (AA)

1-Ethyl-3-(3-dimethylaminopropyl)carbodiimide (EDC) and N-hydroxysuccinimide (NHS) were separately dissolved in water (2 mg/mL). AA was also dissolved in water (1 mg/mL). The EDC (200 μL), NHS (200 μL), and AA solutions (200 μL) were added to the Pdot solution (5 mL). After the addition, the solution was gently shaken for 24 h to prevent Pdot aggregation. The mixture was purified by dialysis for 24 h using a dialysis membrane with a MW cut off (MWCO) of 2000. The various AA-coated Pdots were denoted as BPdots@AA, GPdost@AA, and RPdots@AA originating from BPdots, GPdots, and RPdots, respectively.

### 2.8. Pdot-Embedded Hydrogel

NIPAM, *N*,*N*’-methylenebisacrylamide (BIS), and sodium dodecyl sulfate were dissolved in aqueous Pdots@AA solution (5 mL) under argon. The mixture was heated to 70 °C and potassium persulfate (KPS) was added to the mixture for initiation. The mixture was stirred for 4 h and cooled to room temperature. After filtration through a 0.45 μm filter, the mixture was dialyzed for 72 h using a dialysis membrane with 2000 MWCO. The products were denoted as BPdots@PNIPAM, GPdots@PNIPAM, and RPdots@PNIPAM, which were fabricated from BPdots@AA, GPdots@AA, and RPdost@AA, respectively.

## 3. Results and Discussion

### 3.1. Polymer Synthesis

**Scheme 1:**
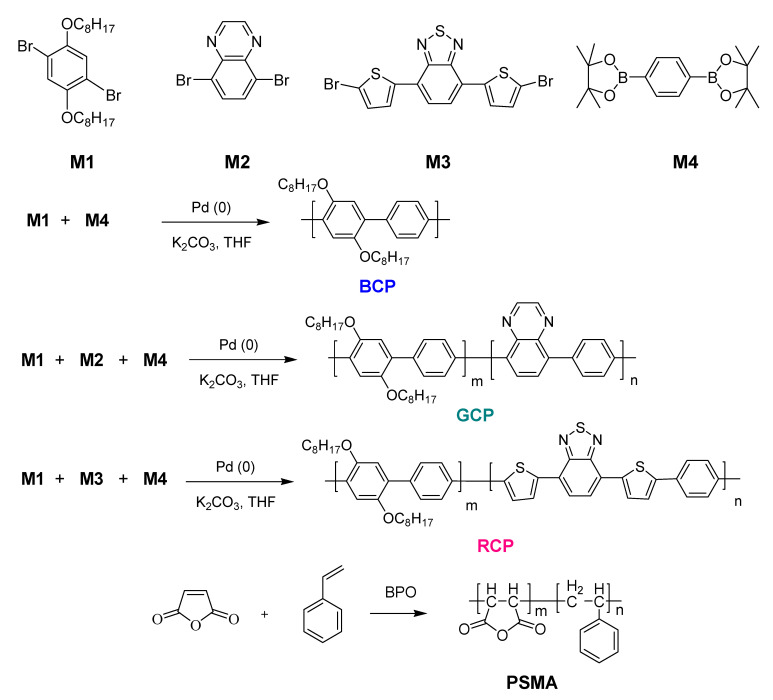
Synthesis of polymers.

**Figure 1:**
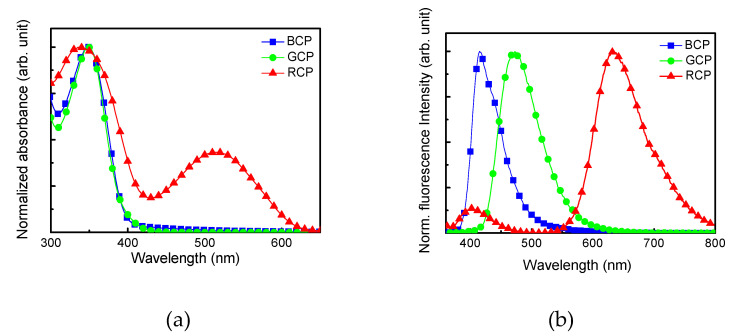
(**a**) UV-vis and (**b**) fluorescence spectra of CP films.

Monomers containing dibromo groups (M1, M2, and M3) were synthesized according to literature methods [33,34,35,36]. BCP, GCP, and RCP were synthesized via the Suzuki coupling reaction in the presence of a Pd catalyst (Scheme 1). PMAS was synthesized by the reaction of styrene and maleic anhydride via conventional radical polymerization with BPO as a radical initiator. The PMAS was used to provide carboxylic acid groups on the Pdot surface, which would be useful for binding to AA. The CPs were soluble in common organic solvents, such as CHCl_3_, DMF, and THF. The CP chemical structures were confirmed with ^1^H and ^13^C NMR and FT-IR spectroscopies, and EA. The molar composition (m:n) of GCP, RCP, and PSMA was determined with EA (Table 1). GPC measurements showed that the number- (M_n_) and weight-average MWs (M_w_) of the polymers ranged from 8200 to 12,100 and from 10,660 to 45,300, respectively, indicating that RCP had a relatively low MW. The UV-vis and fluorescence spectra of the CPs were investigated in THF solutions (Figure S1). The UV-vis absorption and fluorescence emission of BCP in the THF solution were shown at 350 and 415 nm, respectively. GCP exhibited its UV-vis absorption wavelengths at 350 nm and fluorescence was observed at 415 and 490 nm, where the 490 nm fluorescence was attributed to the quinoxaline group in GCP. Like BCP and GCP, RCP showed an absorption wavelength at 345 nm. The emission wavelengths were found at 415 and 630 nm, because of the presence of the bisthienylbenzothiadiazole (BTB) moiety in RCP. Electron-withdrawing groups such as quinoxaline and BTB played an important role in the red shift of UV-vis absorption, showing the green and red fluorescence, respectively. The solid-state of CP presented very similar results to those in the solution (Figure 1). BCP, GCP, and RCP emitted blue, green, and red fluorescence at 415, 475 nm, and 633 nm, respectively, with an identical excitation wavelength at 350 nm, mainly because of the same UV-vis absorption.

### 3.2. Preparation of Polymer Nanoparticle Coated with Allyl Amine

**Scheme 2:**
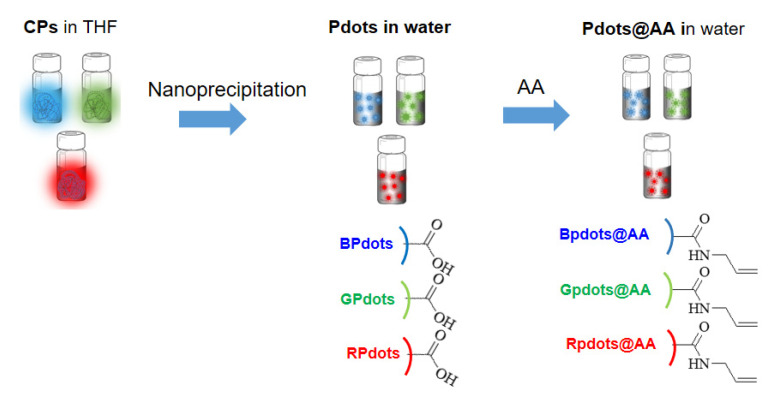
Schematic illustration of Pdot formation via a reprecipitation method and subsequent introduction of AA on Pdot surface to provide Pdots@AA.

**Figure 2:**
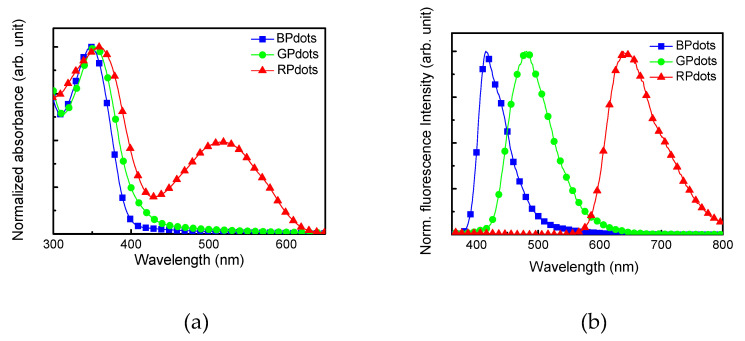
(**a**) UV-vis and (**b**) fluorescence spectra of Pdots in water. Excitation wavelength 350 nm.

Pdots were prepared via the reprecipitation method [24,37]. During Pdot formation, carboxylic acid groups could be generated from the anhydride moieties present on PMAS (Scheme 2). The Pdots exhibited a UV-vis absorption wavelength around 350 nm, with RPdots showing additional absorbance around 500 nm (Figure 2). The Pdots were uniformly dispersed in an aqueous medium without aggregation, but their UV-vis and fluorescence spectra were similar to those of their solid states.

The DLS measurements of Pdots indicated the hydrodynamic diameters to range from 89 to 130 nm and zeta potentials from −11 to −19 mV (Table 2). Because of the presence of the carboxylic acid group on the Pdot surface, the zeta potentials were found to be negative. For further use in linking with NIPAM, AA was introduced to the NP surface by reacting the carboxylic acid moieties of Pdots with AA to form an amide linkage with the aid of EDC and NHS, providing an allyl group (Scheme 2). After the introduction of AA, the NP size remained unchanged, while the zeta potentials of three Pdots@AA changed, because of the transformation of the negatively charged carboxylic acid groups to the allyl groups (Table 2). SEM images of BPdots@AA indicated that the spherical shape of the BPdots was maintained even after AA functionalization (Figure S2). GPdots and RPdots and their AA modified Pdots also showed spherical shapes. The presence of the AA moiety was confirmed by FT-IR and EDS (Figures S3 and S4). In the IR spectra, the characteristic bands of allyl CH=CH_2_ and N-H amide bond were observed at 831 and 1568 cm^−1^, respectively. The EDS mapping images were investigated to determine the N content of the NPs. As expected, N was not found in BPdots, but BPdots@AA showed the presence of N. The UV-vis absorption and fluorescence wavelengths of Pdots@AA were identical to those of Pdots, despite the surface modification with AA (Figure S5). The effect of temperature on the hydrodynamic diameters and fluorescence intensity of the three Pdots@AA was investigated and it was found that those of Pdots@AA were not affected by temperature changes (Figure S6).

### 3.3. Pdot-Embedded Hydrogel

**Scheme 3:**
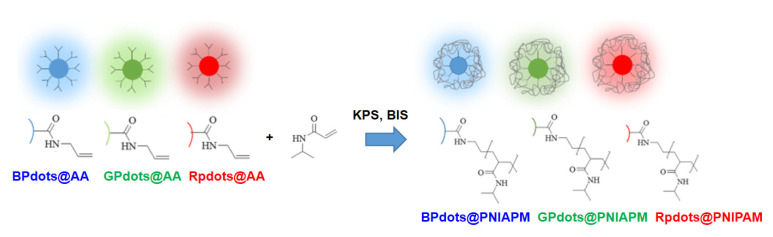
Polymerization of PNIPAM on the surface of Pdots@AA via emulsion polymerization.

**Figure 3:**
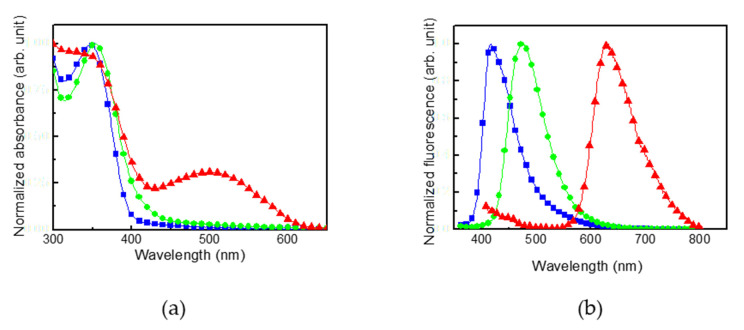
(**a**) UV-vis and (**b**) fluorescence spectra of BPdots@PNIPAM (■), GPdots@PNAIPAM (●), and RPdots@PNAIPAM (▲) in water at 25 °C. Excitation wavelength 350 nm.

Because the Pdots@AA contained allyl functional groups, they could be linked with NIPAM via emulsion polymerization in the presence of the crosslinking agent BIS (Scheme 3). Other hydrogels, including gelatin and chitosan, could be thought of as alternatives to PNIPAM, but they were not considered because the uniform distribution of Pdots and stable immobilization were not possible [38,39,40,41]. The morphology of BPdots@PNIPAM was investigated with SEM and TEM. The spherical shape of BPdots@AA was changed to an aggregated hydrogel shape, indicating that the Pdots were embedded in the PNIPAM hydrogel (Figure S7). Three Pdots@PNIPAM showed an increase in hydrodynamic diameters and the zeta potentials became close to zero, mainly because of the PNIPAM hydrogel layer (Table 3). The UV-vis absorption and fluorescence spectra of the Pdots@PNIPAM indicated the same absorption wavelength of 350 nm with three different emission wavelengths of red, green, and blue (RGB) colors according to the kinds of embedded Pdots (Figure 3).

**Figure 4:**
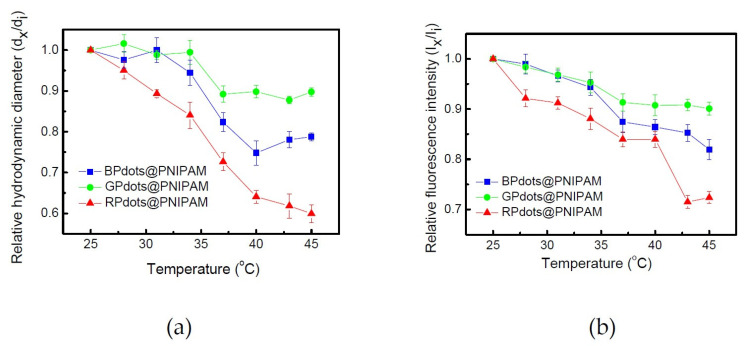
Changes in the (**a**) hydrodynamic diameters and (**b**) fluorescence intensities of Pdots@PNIPAM in aqueous solution upon increase in temperature. d_i_ and d_x_ correspond to hydrodynamic diameters at 25 °C and at elevated temperature, respectively. I_i_ and I_x_ correspond to fluorescent intensities at 25 °C and at elevated temperatures, respectively. Excitation wavelength 350 nm.

**Figure 5:**
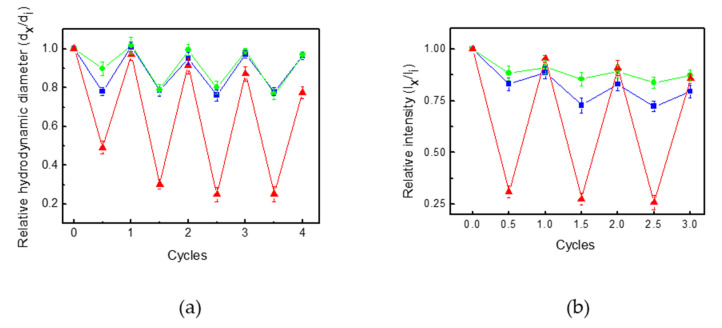
Changes in the (**a**) hydrodynamic diameters and (**b**) relative fluorescence intensities (I_x_/I_i_) of BPdots@PNIPAM (■), GPdots@PNAIPAM (●), and RPdots@PNAIPAM (▲) in aqueous solution over repeated heating (45 °C) and cooling (25 °C) cycles. d_i_ and d_x_ correspond to hydrodynamic diameters at 25 °C and at elevated temperatures, respectively. I_i_ and I_x_ correspond to fluorescent intensities (at 415 nm for BPdots@PNAIPAM; 475 nm for GPdots@PNAIPAM; 625 nm for RPdots@PNAIPAM) at 25 °C and at elevated temperature, respectively.

To elucidate the effect of temperature on the volume change of Pdots@PNIPAM, the LCST behavior of Pdots@PNIPAM was investigated via DLS measurements (Figure 4). The hydrodynamic diameters of Pdots@PNIPAM decreased with the increase in temperature, irrespective of the kinds of Pdots, with an abrupt decrease at about 34 °C, which is the LCST of PNIPAM, induced by the PNIPAM shrinking (Scheme S1). The fluorescence intensity of three Pdots@PNIPAM also gradually decreased with an increase in temperature. It was presumed that the shrinkage of PNIPAM polymer chains interrupted the fluorescence emission of Pdots@PNIPAM above LCST. The polymer chain shrinkage resulted in the aggregation of Pdots@PNIAPM, showing an opaque solution. The aggregation and opaqueness were attributed to the fluorescence decrease. The hydrodynamic diameter and fluorescence intensity of Pdots@PNAIPAM could be controlled by temperature in a reversible fashion, in which they showed stable reversibility in the size and fluorescence after several heating and cooling cycles, indicating that Pdots@PNIPAM had thermo reversibility (Figure 5). Because the changes in the hydrodynamic diameter of RPdots@PNIAPM were larger than those of BPdots@PNIAPM and GPdots@PNIPAM, the changes in the fluorescence intensity of RPdots@PNIPAM were more intense. The thermally-controlled fluorescence modulation was observed as highly noticeable for the red fluorescence from in RPdots@PNIAPM (as high as 80%) and the lowest modulation was observed in GPdots@PNIAPM (20%).

## 4. Conclusions

We synthesize BCP, GCP, and RCP with three primary color emissions via the Suzuki coupling reaction and they were fabricated into Pdots with RGB fluorescence colors. The use of PSMA enabled us to surface-functionalize the Pdots with the carboxylic acid group. The carboxylic acid was reacted with the amine group in AA and the allyl group in AA was introduced to each Pdot surface, linking with NIPAM. After embedding Pdots in the PNIPAM hydrogel, the resultant Pdots@PNIPAM exhibited the RGB fluorescence colors according to the corresponding Pdots used. The hydrodynamic diameter of Pdots@PNIPAM decreased with increasing temperature, which was accompanied by a fluorescence decrease. Because Pdots and PNIPAM were covalently linked together, the composite hydrogel showed stable fluorescence modulation. Such shrinkage and fluorescence decrease were reversible over several heating/cooling cycles, indicating that Pdots@PNIPAM had thermo responsibility.

## Figures and Tables

**Table 1:** Molar compositions ^a^ and molecular weights ^b^ of polymers.

	*m*	*n*	M_n_	M_w_	Polydispersity
BCP			11,100	22,100	1.99
GCP	0.89	0.11	10,200	21,100	2.07
RCP	0.63	0.37	8200	10,660	1.30
PMAS	0.47	0.53	12,100	45,300	3.74

^a^ determined with EA. ^b^ determined with GPC.

**Table 2:** DLS Results of Pdots and Pdots@AA.

Source	Hydrodynamic Diameter (nm)		Zeta Potential (mV)	
	Pdots	Pdots@AA	Pdots	Pdots@AA
BCP	89.0 ± 0.03	78.9 ± 0.03	−34.0 ± 0.3	−19.0 ± 0.5
GCP	130 ± 0.01	125.0 ± 0.7	−27.0 ± 0.8	−13.0 ± 0.5
RCP	122.0 ± 0.8	126.0 ± 0.7	−42.0 ± 0.7	−11.0 ± 0.6

**Table 3:** DLS results of Pdots@PNIPAM.

	Size (nm)	Zeta Potential (mV)
BPdots@PNIPAM	104 ± 0.7	−7.5 ± 0.03
GPdost@PNIPAM	126 ± 0.4	−6.0 ± 0.06
RPdots@PNIPAM	175 ± 0.1	−1.38 ± 0.01

## Supplementary Materials

The following are available online at https://www.mdpi.com/article/10.3390/polym13244315/s1, Scheme S1: Changes in the size and fluorescence intensity of Pdots@PNIAPM upon shrinkage of PNIPAM above the LCST, Figure S1: (a) UV-vis and (b) fluorescence spectra of CPs in THF. Excitation wavelength 350 nm, Figure S2: SEM images of (a) BPdots and (b) BPdots@AA, Figure S3: FT-IR spectra of BPdots (lower) and BPdots@AA (upper), Figure S4: EDS mapping images of (a) BPdots and (b) BPdots@AA, Figure S5: (a) UV-Vis and (b) fluorescence spectra of Pdots@AA in water. Excitation wavelength 350 nm, Figure S6: (a) Effect of temperature on the hydrodynamic diameters of all Pdot@AA in aqueous solution determined by DLS. di and dx correspond to hydrodynamic diameters at 25 °C and at elevated temperature, respectively. (b) Effect of temperature on the relative fluorescent intensity (Ix/Ii) of Pdots@AA in aqueous solution. Excitation wavelength 350 nm. Ii and Ix correspond to fluorescent intensity at 25 °C and at elevated temperature, respectively, Figure S7: (a) SEM and (b) TEM images of BPdots@PNIPAM.
