# Supplementary material for: Fluorescence Modulation of Conjugated Polymer Nanoparticles Embedded in Poly(N-Isopropylacrylamide) Hydrogel
Source: Polymers (Basel). 2021 Dec 9;13(24):4315. doi: 10.3390/polym13244315 (PMC8706719; doi:10.3390/polym13244315)
Supplement: Supplementary file 1 [file polymers-13-04315-s001.zip › polymers-1419088-supplementary.pdf]

Supporting Information

for

Fluorescence modulation of conjugated polymer nanoparticles embedded in poly(N-isopropylacrylamide) hydrogel

Ho Namgung,[+] Seonyoung Jo,[+] Taek Seung Lee*

Organic and Optoelectronic Materials Laboratory, Department of Organic Materials Engineering, Chungnam National University, Daejeon 34134, Korea

[+]These authors contribute equally.

*Corresponding author: TSL (tslee@cnu.ac.kr)

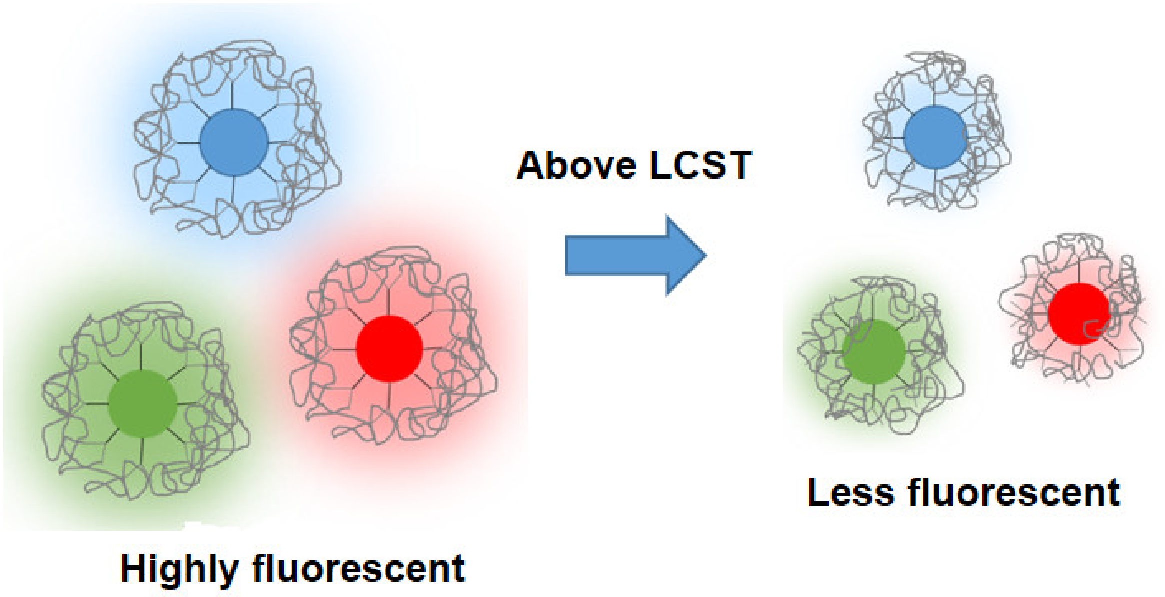

Scheme S1. Changes in the size and fluorescence intensity of Pdots@PNIAPM upon shrinkage of PNIPAM above the LCST.

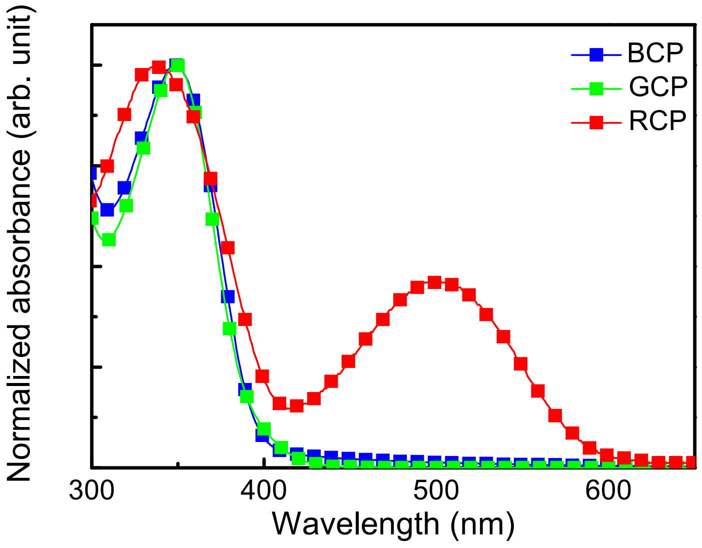

(a)

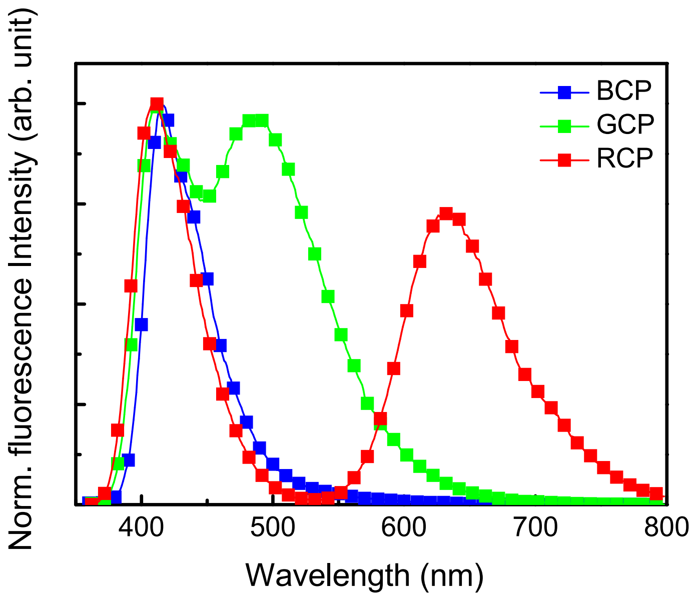

(b)

Figure. S1. (a) UV-vis and (b) fluorescence spectra of CPs in THF. Excitation wavelength 350 nm.

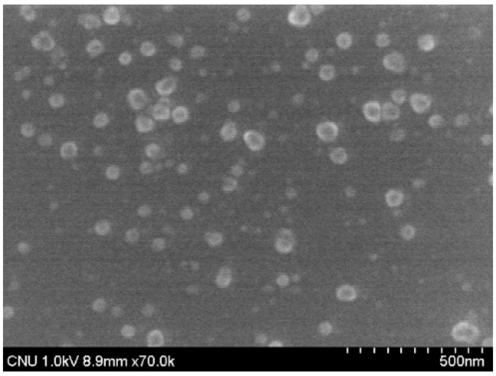

(a)

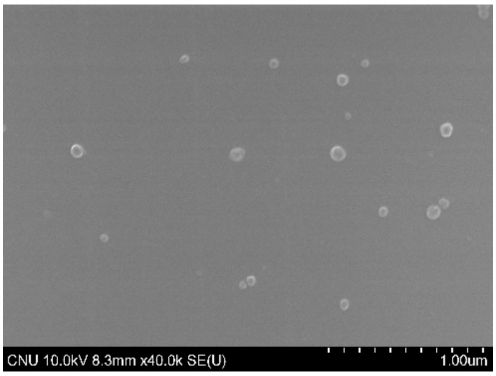

(b)

Figure. S2. SEM images of (a) BPdots and (b) BPdots@AA.

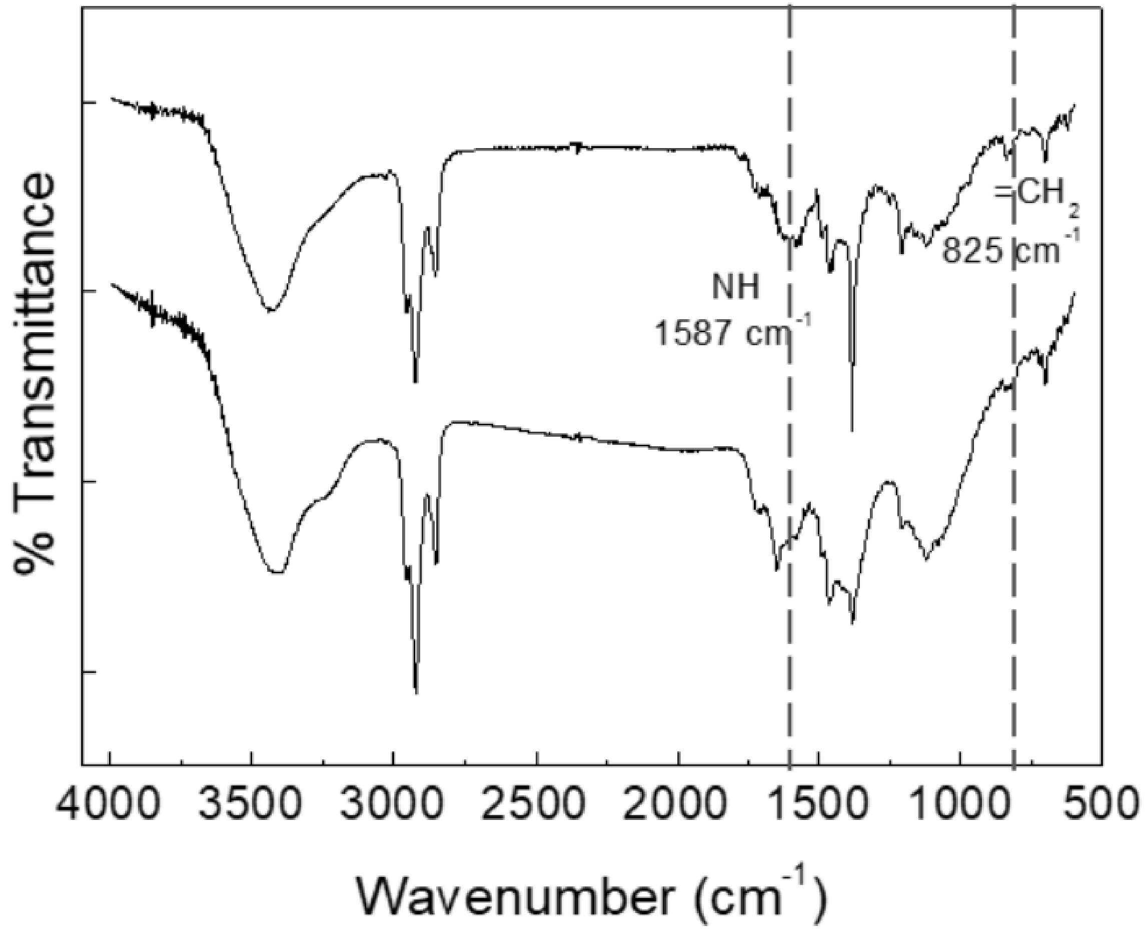

Figure. S3. FT-IR spectra of BPdots (lower) and BPdots@AA (upper).

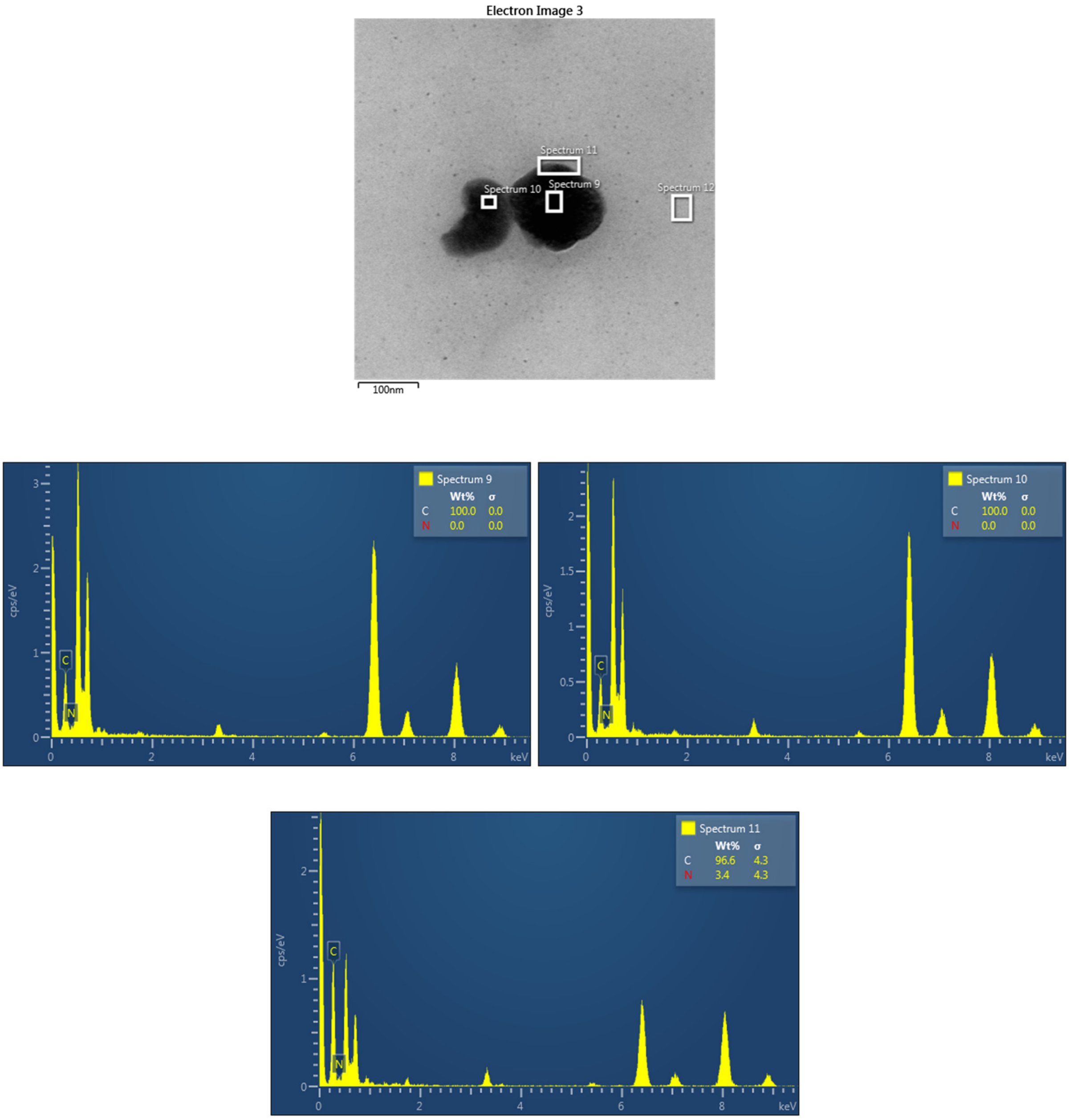
Electron Image 3
Spectrum 11
Spectrum 10
Spectrum 9
Spectrum 12
100nm
Spectrum 9
Wt% σ
C 100.0 0.0
N 0.0 0.0
cps/eV
keV
Spectrum 10
Wt% σ
C 100.0 0.0
N 0.0 0.0
cps/eV
keV
Spectrum 11
Wt% σ
C 96.6 4.3
N 3.4 4.3
cps/eV
keV

(a)

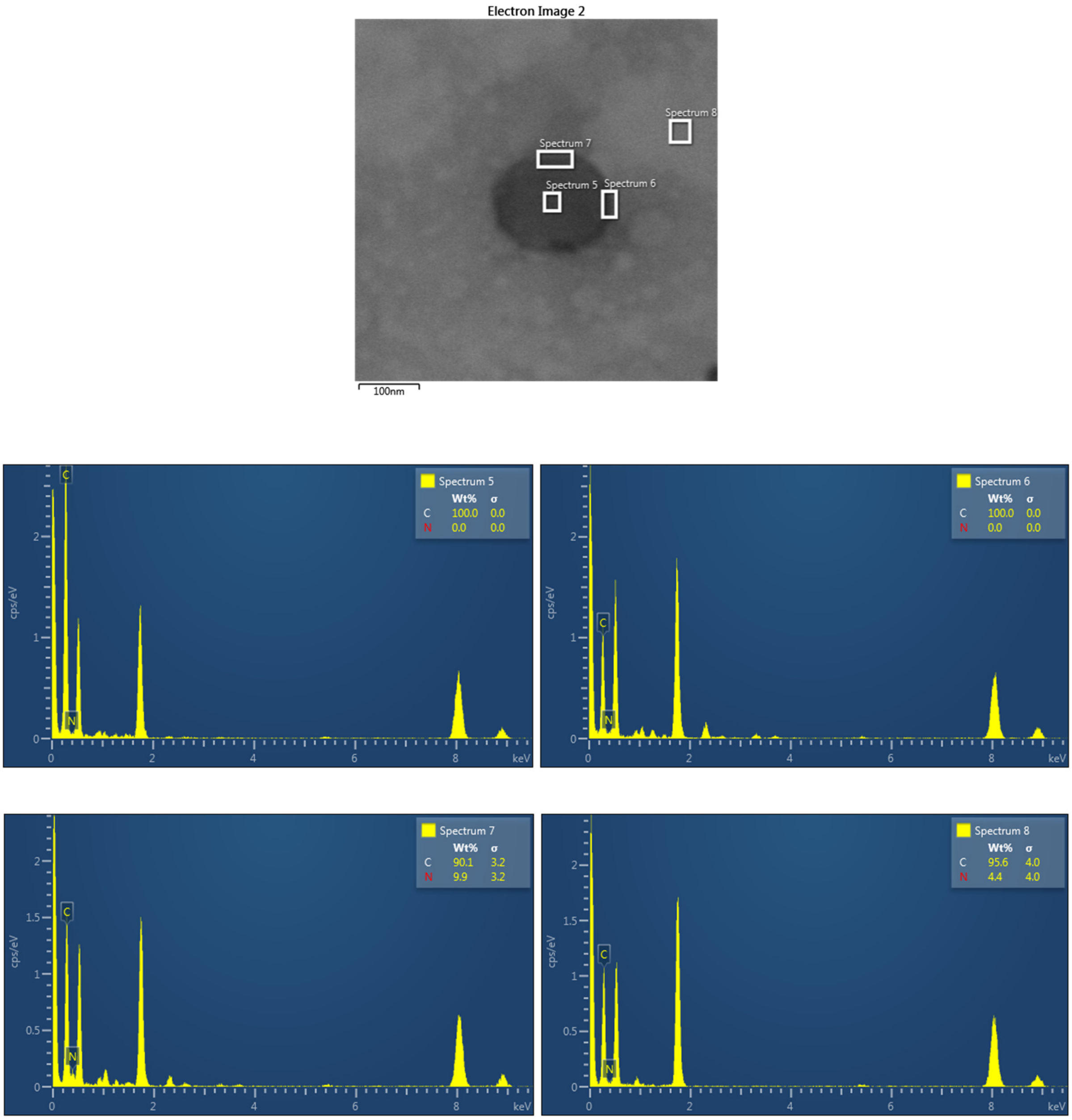

(b)

Figure. S4. EDS mapping images of (a) BPdots and (b) BPdots@AA.

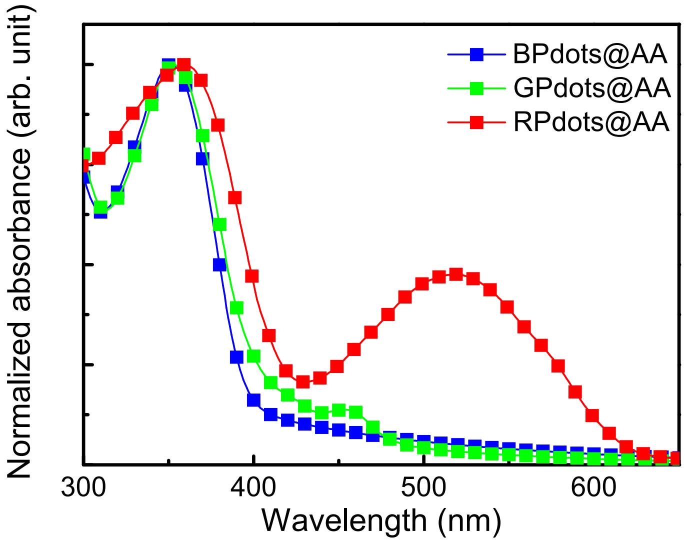

(a)

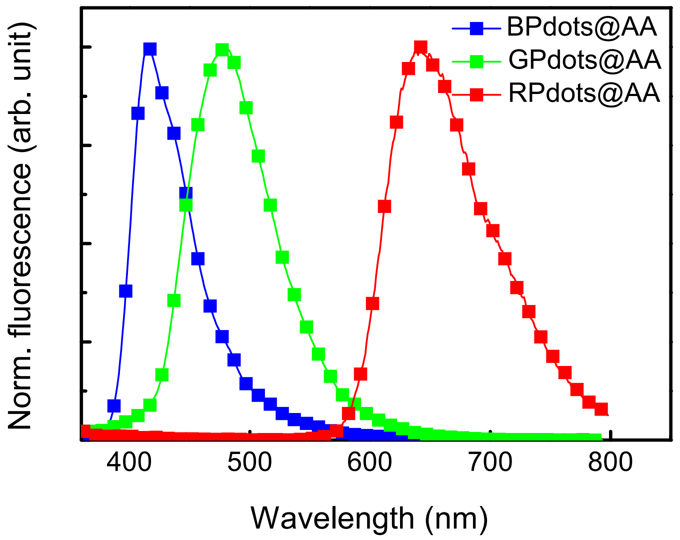

(b)

Figure. S5. (a) UV-Vis and (b) fluorescence spectra of Pdots@AA in water. Excitation wavelength 350 nm.

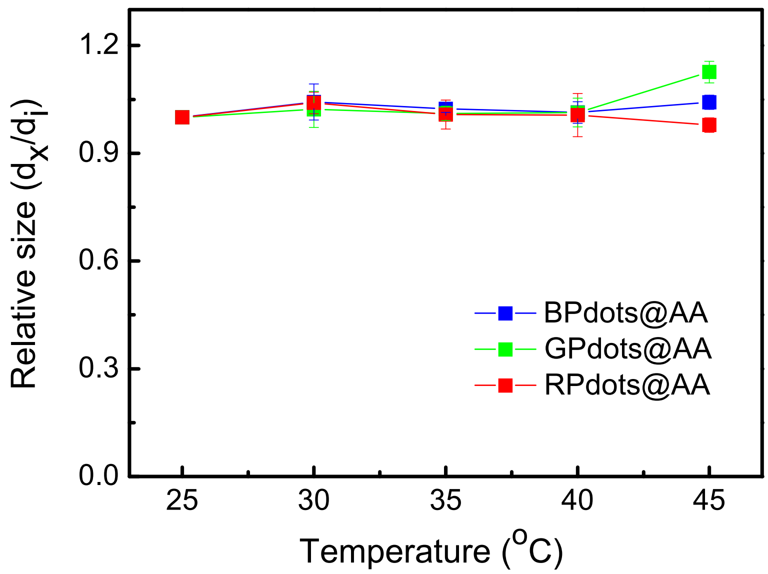

Relative size (d_X/d_i)
1.2
0.9
0.6
0.3
0.0
25
30
35
40
45
Temperature (^oC)
BPdots@AA
GPdots@AA
RPdots@AA

(a)

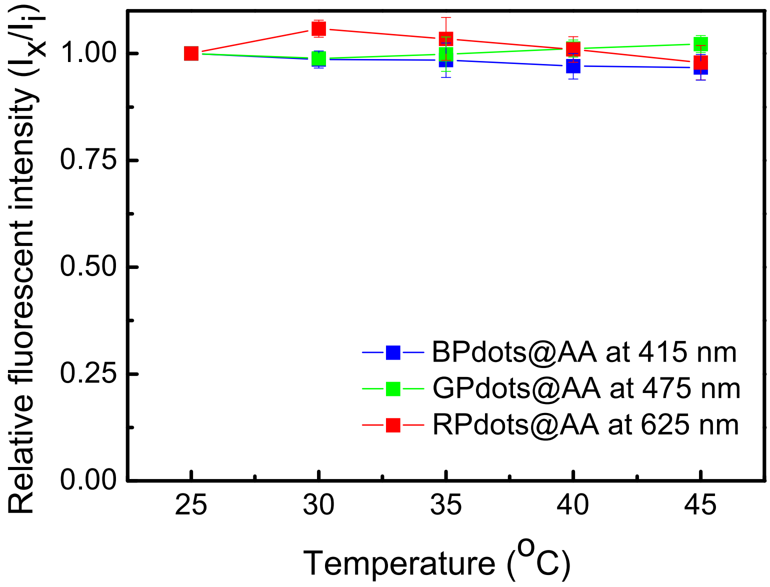

Relative fluorescent intensity (I_X/I_i)
1.00
0.75
0.50
0.25
0.00
25
30
35
40
45
Temperature (^oC)
BPdots@AA at 415 nm
GPdots@AA at 475 nm
RPdots@AA at 625 nm

(b)

Figure. S6. (a) Effect of temperature on the hydrodynamic diameters of all Pdot@AA in aqueous solution determined by DLS. d_i and d_x correspond to hydrodynamic diameters at 25 °C and at elevated temperature, respectively. (b) Effect of temperature on the relative fluorescent intensity (I_x/I_i) of Pdots@AA in aqueous solution. Excitation wavelength 350 nm. I_i and I_x correspond to fluorescent intensity at 25 °C and at elevated temperature, respectively.

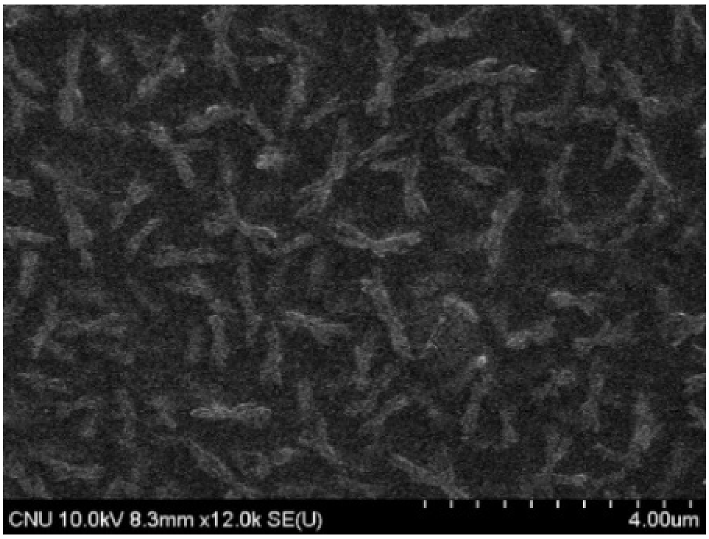

(a)

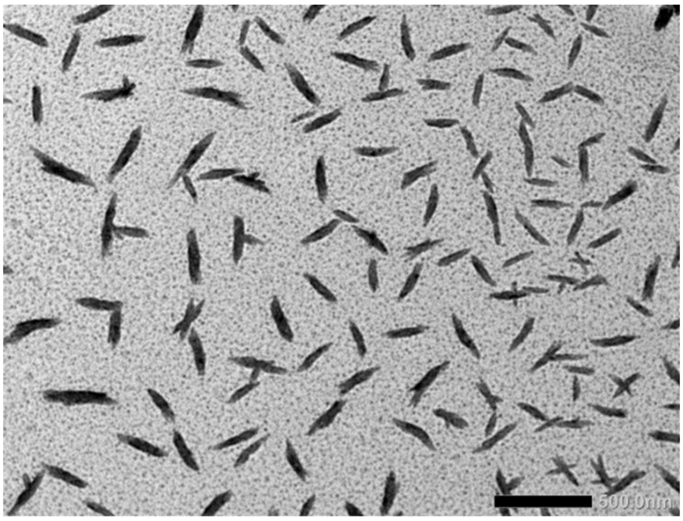

(b)

Figure. S7. (a) SEM and (b) TEM images of BPdots@PNIPAM.
